# Laboratory Evaluation of Asphalt Binders Containing Styrene-Butadiene-Styrene (SBS) and Processed Oil

**DOI:** 10.3390/ma16031235

**Published:** 2023-01-31

**Authors:** Navid Hemmati, Shyaamkrishnan Vigneswaran, Hyun Hwan Kim, Moon-Sup Lee, Soon-Jae Lee

**Affiliations:** 1 Texas State University, San Marcos, TX 78666, USA; 2 Korea Institute of Civil Engineering and Building Technology, Goyang-si 10223, Republic of Korea

**Keywords:** processed oil, SBS, viscosity, stiffness

## Abstract

The study presents an experimental evaluation to improve the resistivity of binders with “Styrene-Butadiene-Styrene” (SBS) and “Processed oil” by studying the physical properties, rheology, and cracking. For this experiment, PG 64-22 was mixed with SBS at different percentages of 5%, 10%, and 15% by weight of the original binder with two processed oil contents of 6% and 12% by weight of the binder. Laboratory tests have been conducted at various high, medium, and low temperature ranges to evaluate their properties. The processed oil polymer modified asphalt (PMA) binder is artificially aged in both the short and long-term using a Rolling Thin Film Oven (RTFO) and a Pressure Aging Vessel (PAV). The Superpave testing method was performed on modified binders using a Rotational Viscometer (RV), Dynamic Shear Rheometer (DSR), and Bending Beam Rheometer (BBR). The results of this study illustrate (1) The addition of SBS leads to higher viscosity, but the co-modification of asphalt binder with the processed oil shows a significant modulation of the viscosity value. (2) In addition, processed oil reduced the resistance to rutting, but the addition of SBS significantly improved the rutting resistance of the asphalt binder. (3) The addition of SBS and processed oil improved the value of G sin *δ*, notably. (4) According to BBR, it has been shown that the addition of SBS in addition to the processed oil improves the stiffness values of modified asphalt binders.

## 1. Introduction

Asphalt binder is a commonly used material in the paving industry. However, with the increased development of cities and countries worldwide, asphalt pavement is becoming more prone to cracking, rutting, moisture damage, and other degradation phenomena under dynamic loads and in extreme weather conditions. These issues can reduce the quality and safety of roads, leading to the need for additional maintenance and reconstruction. As a byproduct of refineries, asphalt is a complex material with unique viscoelasticity due to its high content of asphaltenes and resins [1]. This viscoelasticity makes it an ideal mixture with aggregates for use in different load-bearing situations. It is important to note that the viscoelasticity of asphalt is only effective within a certain temperature range. At too high or too low temperatures, asphalt binder is more likely to experience defects and reduced durability [2]. Asphalt binders exhibit both linear and nonlinear viscoelastic behavior based on the levels of stress and strain they experience [3]. The viscoelasticity of asphalt decreases significantly at low temperatures, which can lead to thermal cracking in older asphalt binders that have higher stiffness [4]. A more in-depth study of the molecular structure of bitumen reveals that the glass transition occurs at a low temperature, resulting in time-dependent hardening and higher stiffness [5]. Older asphalt binders may also experience an increase in viscosity due to the transition from saturates to asphaltenes [6]. Researchers have studied and applied various methods of modifying asphalt mixes to improve their resistance to defects for decades. However, it is important to carefully consider the choice of modifiers given the significant differences between laboratory conditions and real-world implementation. Modifying the asphalt with different materials can increase its viscosity and resistance [7]. Commonly used modifiers in the asphalt industry include styrene-butadiene-styrene (SBS), crumb rubber modifier (CRM), styrene-isoprene-styrene (SIS), styrene-butadiene rubber (SBR) and polyethylene (PE) [7,8]. These days, not only copolymer additives but also nano clay are gathering reputation, such as montmorillonite [9]. Styrene butadiene styrene (SBS) is a copolymer that is commonly used as a modifier for asphalt binders to improve the adhesive properties of the binder. Some co-modifiers such as SBS form a cross-linked network that improves the flexibility, elasticity, and durability of the binder [10]. This allows the asphalt to better resist cracking, rutting, and other forms of distress, which can prolong the life of the pavement. Additionally, SBS also improves the adhesive properties of the asphalt and viscosity consequently, allowing it to better bind to aggregates and form a stronger and more durable road surface [11].

**Figure 1:**
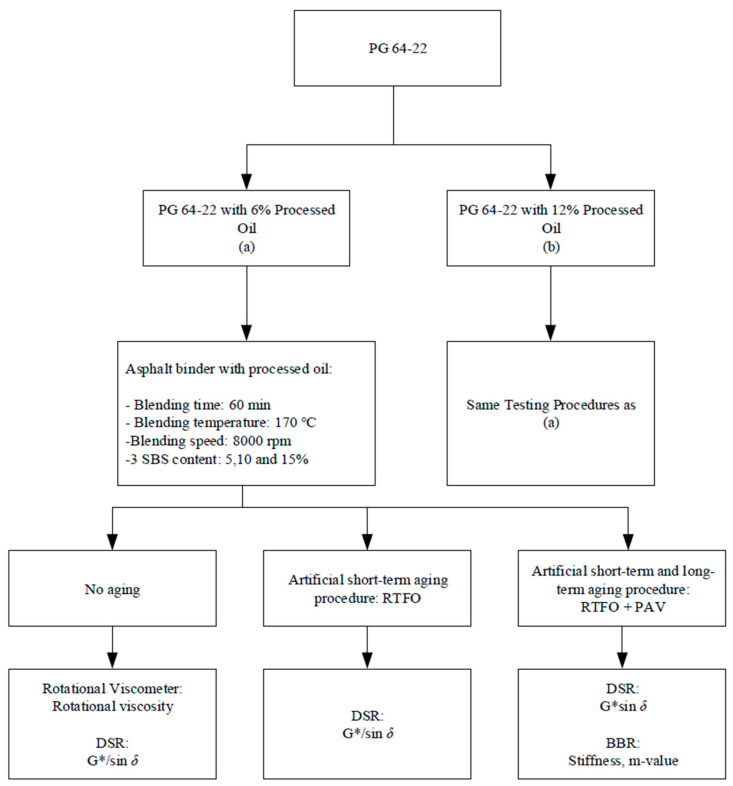
Flow chart of experimental design procedures.

To control the excessively high increase in viscosity, certain auxiliary modifiers, such as oil compounds, can be used to restore the basic physical properties of high-viscosity asphalt and improve the low-temperature performance of the asphalt binder. However, this may affect high-temperature performance [12]. Petroleum compounds, due to their high permeability and solubility in bitumen, can reduce the dynamic viscosity of the asphalt [13,14]. Additionally, applying these compounds to mature asphalt binders can rejuvenate the binder, resulting in decreased stiffness due to viscoelastic recovery [15]. Processed oil, a byproduct of refineries, is effective at reducing the viscosity of bitumen and improving its handling and processability. Treated oils can also be applied and have been shown to reduce viscosity and stiffness due to the high permeability of their molecules and the alteration of asphaltene chains when mixed at high temperatures [16]. Decreased stiffness means less thermal cracking, leading to less maintenance and modification needs. Not only just decreasing the stiffness and viscosity, but higher oil contents yield more fluid binders with better stress-relaxing properties [17]. However, there have been relatively few studies on the effect of processed oils in asphalt mixes, which motivated this study to investigate the performance of asphalt binders co-modified with SBS and processed oils. To understand the resistance to rutting, thermal cracking, and fatigue cracking in modified bituminous binders, the rheological properties of bituminous binders modified with SBS and processed oil was studied after both short-term and long-term artificial aging. Short-term aging was conducted in a rolling thin film oven (RTFO), while long-term aging was conducted in a pressure aging vessel (PAV). To assess high-temperature performance, the viscosity of the asphalt binder in its original state was evaluated using a rotational viscometer (RV) test within a temperature range of 135 °C to 180 °C at 15° intervals. To further evaluate the high temperature and rutting resistance performance of asphalt binders, dynamic shear rheometer (DSR) tests were performed under baseline conditions, short-term after RTFO, and long-term after RTFO + PAV. To assess low-temperature resistance, bending beam rheometer (BBR) tests were performed on long-term aged binder samples at −12 °C and −24 °C. The experimental design flowchart is shown in Figure 1.

## 2. Experimental Design

### 2.1. Materials

In this study, one of the most commonly used asphalt binders in the industry, PG64-22, was modified as a base binder with SBS and processed oil. Table 1 shows the properties of the base adhesive. SBS, as one of the most common modifiers in the industry, was used as the primary modifier in this study, known for its ability to increase viscosity and improve rutting resistance. Table 2 shows the characteristics of SBS. Processed oil is a byproduct of petroleum refining that is often used in lubrication applications. In this study, it was used as an auxiliary modifier to improve excessive viscosity and moderate low-temperature performance, as well as facilitate handling and processability. Table 3 illustrates the properties of processed oil.

### 2.2. Production and Preparation of SBS-Processed Oil-Modified Asphalt Binder

Samples were prepared using the wet process by adding SBS and processed oil directly to the base binder at a temperature of 170 ± 5 °C. Weighed processed oils (6 and 12%) were added and mixed with base asphalt binders using a high-speed homogenizer at 8000 rpm for 10 min while manually monitoring the temperature to ensure that it did not exceed the boiling point and cause loss of basic binder properties. And then prepared SBS powder with four contents (0, 5, 10, and 15%) was added into processed oil-modified binders. For SBS modification, the same mixing condition was applied with processed oil. The modifier and co-modifier materials were sourced from the same batch to ensure consistency. The samples were then subjected to two stages of artificial aging: short-term and long-term. Short-term aging was performed in a rolling thin film oven (RTFO) at 163 °C for 85 min, followed by long-term aging in a pressure aging vessel (PAV) at 100 °C for 20 h at 2.1 MPa. The experiments in the experimental design flowchart were conducted to examine the samples and study the performance of SBS-modified asphalt binders and processed oil.

#### 2.2.1. Basic Characteristics Tests

Rotational viscosity tests were conducted through a temperature range of 135 °C to 180 °C with 15 °C intervals and a spindle speed of 20 rpm to evaluate the workability of the asphalt binder which was derived from the relation between fluid shear and spindle radius and rotation speed. The prepared sample was reduced to 10.5 g and tested using a 27-gauge cylindrical spindle. Data was collected for 20 min at 1-min intervals.

To further assess viscosity, the complex shear modulus (G*) and phase angle sine (sin*δ*) were measured at 82 °C by testing via a DSR device, from which G*/sin *δ* was calculated. By measuring G* and sin *δ* after RTFO + PAV at 25 °C and calculating G*/sin *δ* accordingly, the fatigue cracking resistance of the asphalt binder was evaluated. Binder stiffness was measured at −12 °C and −24 °C using BBR equipment (Canon, Huntington, NY, USA) according to AASHTO T 313 specification. The specimen mold was prepared and cast to the required size (125 × 6.35 × 12.7 mm^3^) and immersed in a methanol bath in the BBR to measure the creep stiffness of the specimen under a load mass of 100 g for 60 s.

#### 2.2.2. Statistical Analysis Method

In this study, statistical analysis was performed using analysis of variance (ANOVA) and Fisher’s Least Significant Difference (LSD) comparison with an alpha level of 0.05, calculated using Procedures for the Social Sciences (SPSS). The variables analyzed were processed oil at 0%, 6%, and 12% and SBS at 0%, 5%, 10%, and 15%. ANOVA was used to identify significant differences between sample means. The significance level for this study was set at 95% (alpha = 0.05), indicating a 95% probability of obtaining the true value. The LSD was calculated after identifying unequal means among the samples and used to determine the difference between the two sample means. Sample comparison was performed after calculating the LSD, and population means were determined to be statistically different when the difference between the two sample means was greater than or equal to the LSD.

## 3. Results and Discussion

### 3.1. Rotational Viscosity

**Figure 2:**
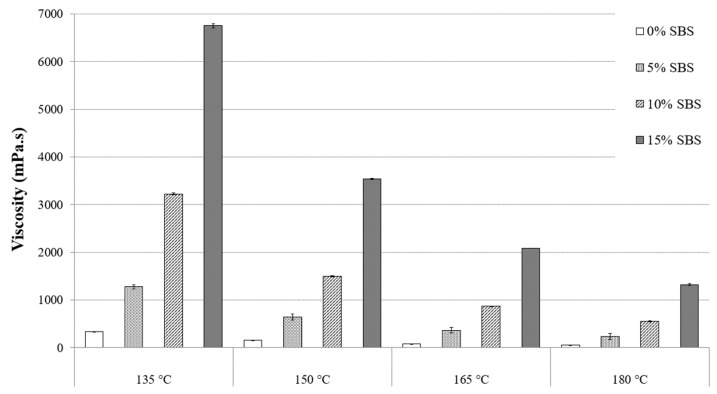
Viscosity of PG 64-22 binders with 6% processed oil as a function of SBS content.

**Figure 3:**
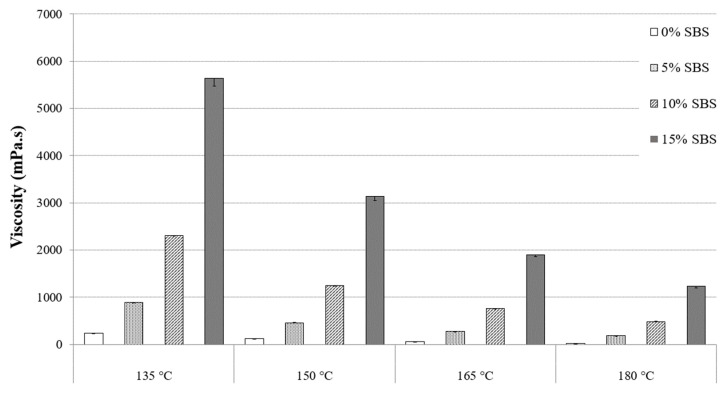
Viscosity of PG 64-22 binder with 12% processed oil as a function of SBS content.

To optimize the quality of asphalt pavement, mixing conditions, such as temperature and time, must be accurately determined to improve handling, workability, aggregate encapsulation, and uniform compaction [27]. Figure 2 and Figure 3 show the results of standard RV tests for PG 64-22 asphalt binder modified with 6% and 12% processed oil and 0%, 5%, 10%, and 15% SBS and processed oil at 135 °C, 150 °C, 165 °C, and 180 °C. Table 4 and Table 5 are showing the viscosity values for both modified asphalt binders with 6% and 12% processed oil, containing 0%, 5%, 10%, and 15% SBS. The modified asphalt binders with processed oil and without any content of SBS are considered to be control binders to be compared with the other samples which are containing SBS. Figure 2 shows the modified asphalt binder with 6% processed oil and increasing viscosity values with the addition of 0%, 5%, 10%, and 15% SBS at each temperature range. At 135 °C, viscosity values of 335, 1281, 3225, and 6756 mPa⋅s were recorded for 0%, 5%, 10%, and 15% SBS, respectively. In comparison, Figure 3 shows viscosity values of 243, 887, 2306, and 5637 mPa⋅s for the modified asphalt binder containing 12% processed oil and SBS at the same temperature and concentration. These results indicate that increasing the processed oil content from 6% to 12% caused a decrease in viscosity values of 27%, 30%, 28%, and 16% due to the high-rate penetration of processed oil and alteration of asphaltene chains in asphalt binder molecules. Comparing the two figures shows a similar trend for the rest of the viscosity values. At 150 °C, a comparison between Figure 2 and Figure 3 with 0%, 5%, 10%, and 15% SBS shows a decrease in viscosity values of 26%, 28%, 16%, and 11% due to an increase in processed oil from 6% to 12%. A similar pattern can be seen at 165 °C, and the effect of processed oil can be seen at 180 °C, with a decrease in viscosity values of 50%, 19%, 12%, and 6% for 0%, 5%, 10%, and 15% SBS, respectively, due to an increase in processed oil content from 6% to 12%. While the SBS modifier significantly increases G*/Sin *δ* [27], the addition of processed oil leads to a decrease in viscosity due to its higher permeability and the reorganization of asphaltene nano-aggregates during high-temperature mixing, resulting in improved handling and workability. Therefore, the 12% processed oil level had a greater impact on viscosity compared to the 6% processed oil. According to Asphalt Institute standards, modified asphalt binders with 6% and 12% processed oil and 0%, 5%, and 10% SBS had viscosities less than 3000 mPa⋅s at 135 °C [27]. However, both modified asphalt binders with 6% containing 10% and 15% SBS and modified asphalt binder with 12% processed oil containing 15% SBS showed higher values of viscosity and exceeded the standard value of 3000 mPa⋅s (AASHTO M332), but the addition of processed oil showed a significant effect on the workability and handling of the samples. The application of processed oil may lower the placement temperature due to the decrease in viscosity. On the other hand, based on the ease of handling and enhancement of workability, the mixing design of the batches can be modified.

Table 6 and Table 7 show the statistical significance of the variation in viscosity as a function of processed oil and SBS content. The results indicate that increasing the content of processed oil significantly impacts the changes in viscosity due to temperature changes during SBS conjugation. The addition of processed oil and SBS causes a significant shift from the control binder, demonstrating the effect of SBS on viscosity as the processed oil infiltrates the molecular chain and enhances the modified asphalt binder’s workability and handling.

### 3.2. Rutting Properties

**Figure 4:**
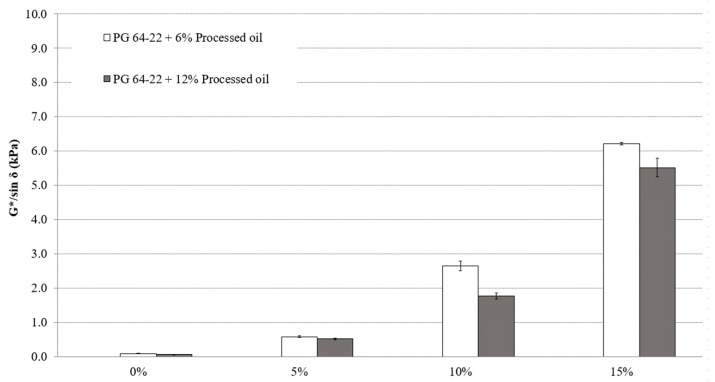
G*/sin *δ* of PG 64-22 binders with processed oil as a function of SBS content at 82 °C.

As per super pave specifications [27], higher G*/sin *δ* in the DSR test leads to improved resistance to high-temperature rutting defects. The G*/sin *δ* evaluation was performed in the original state and after short-term aging (RTFO) at 82 °C. Figure 4 displays the results of the DSR test in the original state. The processed oil, however, decreases the viscosity of binders. The results show that both 6% and 12% processed oil modified with SBS content of 5%, 10%, and 15% have improved rutting resistance due to the upward trend in G*/sin *δ* values. Figure 4 illustrates that for modified asphalt binders containing 6% processed oil and 0%, 5%, 10%, and 15% SBS, the G*/sin *δ* values are 0.085, 0.578, 2.64, and 6.2 kPa, respectively. For modified asphalt binders containing 12% processed oil and 0%, 5%, 10%, and 15% SBS, the G*/sin *δ* values are 0.06, 0.51, 1.76, and 5.5 kPa, respectively. Comparing 6% and 12% processed oil content, a higher processed oil content moderates the increase in G*/sin *δ*. 12% processed oil showed slightly lower values at all SBS contents rather than 6% oil content. This indicates that the higher processed oil content in PMA binders does not improve the rutting resistance even though it was positive in workability with lower viscosity.

Table 8 demonstrates significant changes in G*/sin *δ* in relation to SBS and processed oil content. For the modified asphalt binder with 6% processed oil, significant changes were observed, apart from the sample containing 5% SBS. The modified asphalt with 12% processed oil also showed significant changes. The results indicate that the addition of SBS conjugating processed oil significantly improves the performance of modified asphalt binders against rutting defects compared to the control asphalt binder, due to the high penetration rate of processed oil and the restructuring of asphaltene molecules.

**Figure 5:**
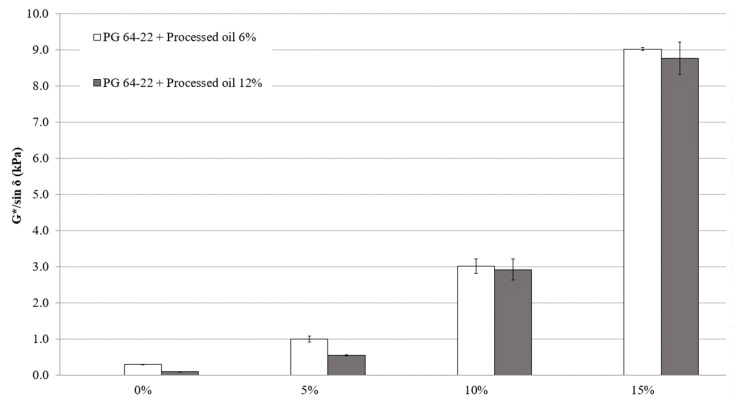
G*/sin *δ* of RTFO aged PG 64-22 binders with processed oil as a function of SBS content at 82 °C.

The G*/sin *δ* values of modified asphalt binders containing 6% and 12% processed oil and various amounts of SBS (0%, 5%, 10%, and 15%) were measured in a short-term aged state. As shown in Figure 5, the G*/sin *δ* values for the modified binders containing 6% processed oil were 0.29, 1.0, 3.0, and 9.0 kPa for the respective SBS amounts. The G*/sin *δ* values for the modified binders containing 12% processed oil were 0.095, 0.54, 2.92, and 8.77 kPa for the respective SBS amounts. Both Figure 4 and Figure 5 demonstrate an increment in G*/sin *δ* when SBS is added to the binders, which indicates that SBS can enhance rutting resistance. However, the increase in processed oil was ineffective in improving the rutting property of short-term aged binders, as shown in the original state. Therefore, considering the effect of processed oil on viscosity, it is thought that the softened property of PMA binders with less rutting resistance is reflected in lower G*/sin *δ* results.

Table 9 presents the effect of adding SBS on the G*/sin *δ* values of two modified asphalt binders with 6% and 12% processed oil. The G*/sin *δ* values for the modified binder containing 6% processed oil exhibited significant changes with the addition of SBS, except for the sample with 5% SBS. Similarly, the modified binder with 12% processed oil displayed significant changes in the G*/sin *δ* values upon the addition of SBS.

### 3.3. Fatigue Cracking

**Figure 6:**
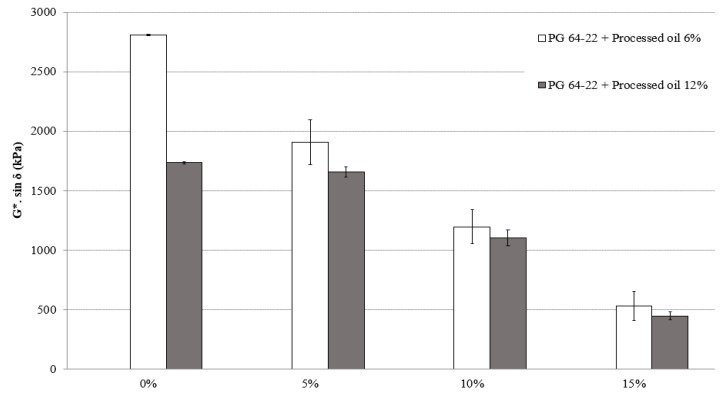
G*sin *δ* of RTFO + PAV aged PG 64-22 binders with processed oil as a function of SBS content at 25 °C.

The fatigue cracking characteristics of asphalt binders after long-term aging (RTFO + PAV) treatment was evaluated using DSR tests at 25 °C. The results, based on G* sin *δ*, are shown in Figure 6 for modified asphalt binders containing 6% and 12% processed oil and various amounts of SBS (0%, 5%, 10%, and 15%). As shown in Figure 6, the addition of SBS to the modified asphalt binders containing 6% processed oil resulted in a decreasing trend in G* sin *δ* values, with decreases of 67%, 62%, and 44% for SBS contents of 5%, 10%, and 15%, respectively. The G* sin *δ* values for the modified binders containing 12% processed oil also showed a decreasing trend, with decreases of 95%, 66%, and 40% for SBS contents of 5%, 10%, and 15%, respectively. The aging process causes the transition of saturates to asphaltene in the asphalt binder molecule structure and increases the viscosity due to the SBS content. SBS cross-linking enhances the asphalt binder’s capacity to spread out energy, which diminishes the concentration of stress at the crack tip and slows the crack’s spread. Furthermore, the cross-linked network of SBS allows the asphalt binder to adjust better to the varying loads and temperatures it encounters on the pavement, further decreasing the possibility of crack initiation and progression. To sum up, SBS cross-linking improves the resistance of the asphalt binder to fatigue cracking by creating a more robust, more flexible, and long-lasting binder that can dissipate energy and adjust to changing loads and temperatures. However, the processed oil has a significant effect on G* sin *δ* because the oil molecules break down during high-temperature mixing and alter the viscosity of the asphalt molecules, resulting in significant changes in the measured values.

Table 10 presents the statistical analysis of the significant changes in the modified asphalt binders containing 6% and 12% processed oil and various amounts of SBS (0%, 5%, 10%, and 15%). The analysis shows that the modified asphalt binder with 6% processed oil exhibits significant changes due to the addition of SBS. The modified asphalt binder with 12% processed oil also shows significant changes with the addition of SBS, except for the change from 0% to 5% SBS, which was not statistically significant.

### 3.4. Low-Temperature Cracking Properties

**Figure 7:**
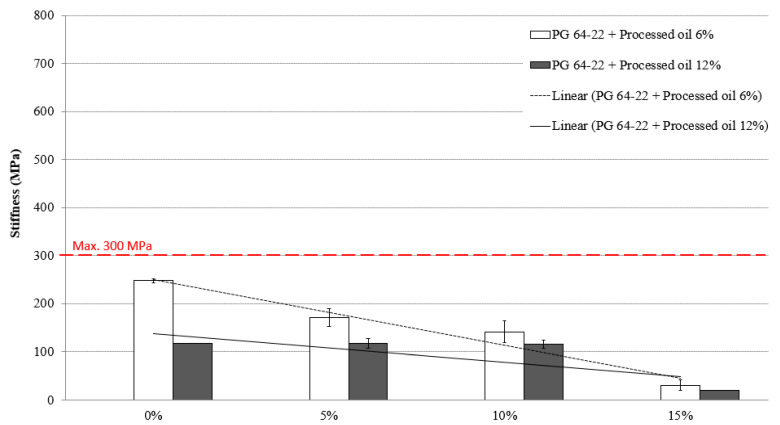
Stiffness of RTFO + PAV aged PG 64-22 binder with processed oil as a function of SBS content at −12 °C.

**Figure 8:**
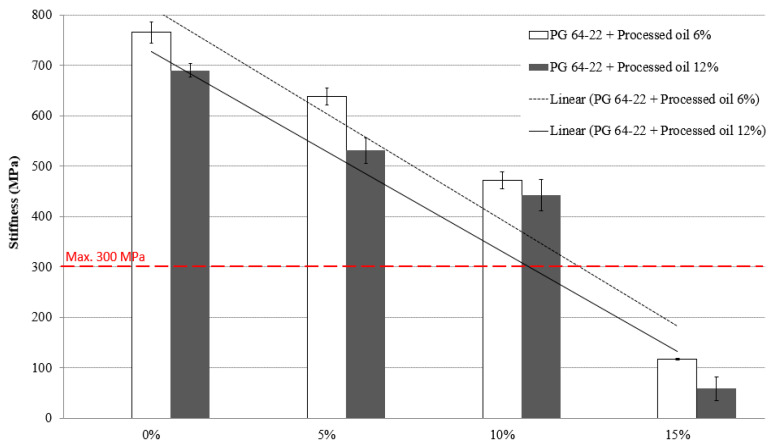
Stiffness of RTFO + PAV aged PG 64-22 binder with processed oil as a function of SBS content at −24 °C.

In accordance with Superpave specifications, the maximum allowed creep stiffness of asphalt binders should not exceed 300 Mpa to avoid thermal cracking at low temperatures. The BBR test was conducted to determine the creep stiffness of modified asphalt binders containing 6% and 12% processed oil and various amounts of SBS (0%, 5%, 10%, and 15%) at −12 °C and −24 °C. The results, presented in Figure 7 and Figure 8, show the creep stiffness values of the modified binders at these temperatures. At −12 °C, the stiffness values for the modified asphalt binders containing 6% processed oil and 0%, 5%, 10%, and 15% SBS were measured as 247, 171, 141, and 30 MPa, respectively. The modified asphalt binders containing 12% processed oil and the same concentrations of SBS had stiffness values of 117, 117, 115, and 19 MPa at −12 °C. When tested at −24 °C, the stiffness values for the modified asphalt binders containing 6% processed oil and 0%, 5%, 10%, and 15% SBS were measured as 765, 638, 472, and 117 MPa, respectively. The modified asphalt binders containing 12% processed oil and 0%, 5%, 10%, and 15% SBS had stiffness values of 690, 531, 442, and 59 MPa at −24 °C. At both temperatures, the stiffness of the asphalt binders containing the same concentrations of SBS decreased significantly with an increase in the processed oil content from 6% to 12%. This demonstrates the positive effect of processed oil on reducing stiffness and improving the resistance of asphalt binders against thermal cracking. The lower viscosity achieved through the penetration of processed oil during mixing and restructuring of asphaltene nano-aggregates leads to lower creep stiffness for the samples containing 12% processed oil compared to the samples containing 6% processed oil.

Table 11 and Table 12 present the significant changes observed in binders modified with 6% and 12% processed oil that contained 0%, 5%, 10%, and 15% SBS. The analysis in Table 9 reveals that the inclusion of 15% SBS leads to significant changes compared to the addition of 5% and 10% SBS. However, the incorporation of processed oil allows for the achievement of creep stiffness lower than 300 MPa in all modified asphalt binders containing 6% and 12% processed oil.

Table 12 represents the creep stiffness results at −24 °C, which illustrates that due to the increase of SBS and processed oil content, the changes are significant and both modifiers play an important role in the decrease of stiffness.

**Figure 9:**
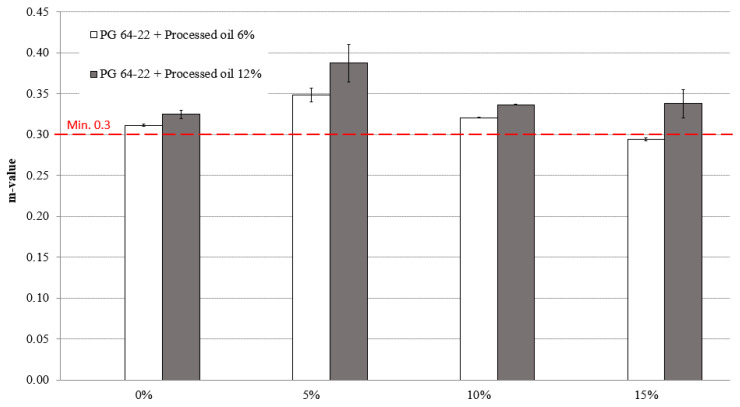
m-value of RTFO + PAV aged PG 64-22 binders with processed oil as a function of SBS content at −12 °C.

**Figure 10:**
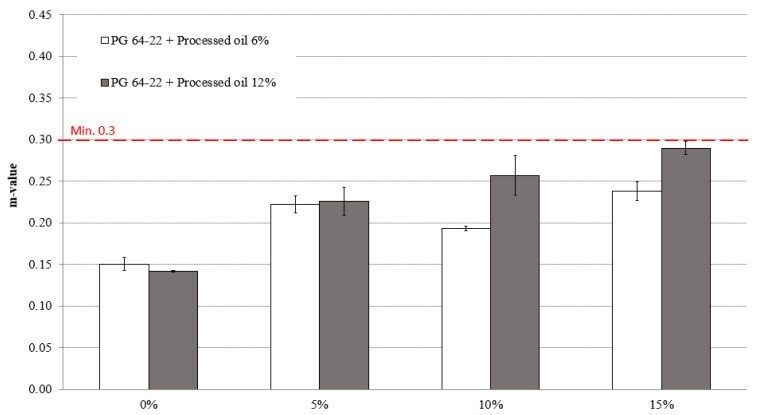
m-value of RTFO + PAV aged PG 64-22 binder with processed oil as a function of SBS content at −24 °C.

Figure 9 and Figure 10 present the m-values of modified asphalt binders containing 6% and 12% processed oil with 0%, 5%, 10%, and 15% SBS at −12 °C and −24 °C. The obtained pattern of creep stiffness values shows a moderate increase in both modified asphalt binders with 6% and 12% processed oil. At −12 °C, the modified asphalt binder with 6% processed oil exhibits m-values of 0.311, 0.348, 0.320, and 0.294 for SBS contents of 0%, 5%, 10%, and 15%, respectively. Similarly, the modified asphalt binder with 12% processed oil displays m-values of 0.325, 0.387, 0.336, and 0.337 for SBS contents of 0%, 5%, 10%, and 15% at −12 °C.

As shown in Figure 10, a more noticeable increase in the pattern of m-values was observed at −24 °C due to the lower temperature compared to −12 °C. The modified asphalt binder with 6% processed oil exhibited m-values of 0.15, 0.222, 0.193, and 0.238 for SBS contents of 0%, 5%, 10%, and 15%. Similarly, the modified asphalt binder with 12% processed oil displayed m-values of 0.141, 0.226, 0.257, and 0.289 for SBS contents of 0%, 5%, 10%, and 15% at −24 °C.

Table 13 and Table 14 illustrate the statistical analysis of m-value changes as a function of processed oil and SBS content. Table 11 shows the significance of differences in m-values between asphalt binders including 0%, 5%, 10%, and 15% SBS with 6% and 12% processed oil. The results for the modified asphalt binder with 6% processed oil show significant changes for all samples except the addition of SBS content from 5% to 10%. In the modified asphalt binder with 12% processed oil, changes in SBS content were significant with the addition of 5%, 10%, and 15% compared to 5%. Changes from 10% to 15% were also insignificant.

Table 14 presents the significance of changes in the m-values of modified asphalt binders containing 6% and 12% processed oil as a function of SBS contents at −24 °C. The modified asphalt binder with 6% processed oil exhibits significant changes in all cases except when comparing 5% and 15% SBS. In contrast, the modified asphalt binder with 12% processed oil shows non-significant changes when comparing 5% SBS with 10% SBS and 10% SBS with 15% SBS. All other changes in the modified asphalt binder with 12% processed oil are significant.

## 4. Summary and Conclusions

The properties of modified asphalt binders containing processed oil and SBS were evaluated by artificially aging original asphalt binders with 6% and 12% processed oil containing 0%, 5%, 10%, and 15% SBS for the short-term and long-term. Rotational viscosity, DSR, and BBR tests were conducted to study the properties of these modified asphalt binders at different concentrations. The following conclusions were drawn based on the results:

1. The addition of processed oil in concentrations of 6% and 12%, combined with different SBS concentrations, decreases the viscosity values of the asphalt binders, improving their handling and workability. This is due to the restructuring of asphaltene nano-aggregates molecules at high mixing temperatures resulting from processed oil addition during modification
2. While the addition of SBS increases the viscosity of asphalt binders, this study found that processed oil has a positive influence on the combination of SBS and the modified asphalt binders meet the majority of Superpave specifications.
3. Even though the addition of SBS positively affected rutting properties with higher G*/sin *δ*, the processed oil showed opposite results. This is considered to have softened the binder with the addition of processed oil and has been reflected in less G*/sin *δ* values.
4. The DSR test was used to evaluate fatigue cracking resistance and showed a significant effect on processed oil. This is attributed to the high penetration rate of processed oil in asphalt binders and the restructuring of asphaltene molecules.
5. The addition of processed oil resulted in a significant decrease in stiffness values. The properties were evaluated at −12 °C and −24 °C and results showed that a higher content of processed oil significantly decreases creep stiffness. The same improvement in m-values was observed due to the viscosity and viscoelasticity improvements resulting from the addition of processed oil in concentrations of 6% and 12%.
6. The use of SBS as a modifier and processed oil as a co-modifier improves the resistance of asphalt binders at high and low temperatures and eases their handling and workability. Further research into the use of processed oil in combination with other modifiers like SBR and LDPE is recommended.
7. The application of processed oil may result in a decrease in the onsite placement temperature due to a decrease in viscosity, and enhance the workability and handling of the asphalt mixtures on one side, while also providing sufficient time for the aggregates to have maximum contact with the asphalt on the other side.

## Figures and Tables

**Table 1:** Properties of base asphalt binder (PG 64-22).

Aging States	Test Properties	Standard	PG 64-22
Unaged	Viscosity @ 135 °C (mPa·s)	AASHTO PP6	538
	G */sin *δ* @ 64 °C (kPa)	AASHTO PP6	2.2
RTFO aged residual	G */sin *δ* @ 64 °C (kPa)	AASHTO PP6	3.4
RTFO + PAV aged residual	G *sin *δ* @ 25 °C (kPa)	AASHTO PP6	4400
	Stiffness @ −12 °C (MPa)	AASHTO PP6	187
	m-value @ −12 °C	AASHTO PP6	0.32

**Table 2:** Properties of SBS.

Parameter	Unit	Value	Method
Density	g/cm^3^	0.94	ISO 2781 [18]
Toluene Solution Viscosity	cSt	13	ASTM D445 [19]
Hardness	Shore A	79	ASTM D2240 [20]
Molecular Weight	g/mol	262.4	
Volatile Matter	%	<0.6	ASTM D1416 [21]
Yellow Index		<6	ASTM D1925 [22]

**Table 3:** Properties of processed oil.

Test Item	Condition	Unit	Result	Standard
Specific gravity	@ 15 °C		1.0049	ASTM D4052 [23]
Flash Point	COC	°C	228	ASTM D 92 [24]
Kinematic Viscosity	@98.9 °C	mm^2^/S	22.59	ASTM D 445
	@100 °C	mm^2^/S	21.53	ASTM D 445
Pour point		°C	+15	ASTM D 97 [25]
Viscosity Gravity Constant			0.9555	ASTM D 2140 [26]
Hydrocarbon type	Ca “aromatic carbon atoms”	%	41.9	ASTM D 2140
	Cn “aliphatic carbon atoms”	%	26.2	ASTM D 2140
	MPa⋅sMPa⋅s “polar carbon atoms”	%	31.9	ASTM D 2140

**Table 4:** Viscosity values for PG 64-22 with 6% processed oil as a function of the SBS content and temperature.

	135 °C	150 °C	165 °C	180 °C
SBS Content	Viscosity (mPa·s)			
0%	335.2	162.25	86.75	50.5
5%	1281.3	643.75	368.75	233.75
10%	3225.0	1500.0	868.75	556.25
15%	6756.3	3543.5	2087.5	1325.0

**Table 5:** Viscosity values for PG 64-22 with 12% processed oil as a function of the SBS content and temperature.

	135 °C	150 °C	165 °C	180 °C
SBS Content	Viscosity (mPa·s)			
0%	243.75	118.75	62.5	25
5%	887.25	462.25	281.25	187.5
10%	2306.25	1250.0	756.25	487.5
15%	5637.5	3137.5	1900.0	1237.5

**Table 6:** Statistical analysis results of viscosity for PG 64-22 with 6% processed oil as a function of SBS content (α = 0.05).

Viscosity		135 °C				150 °C				165 °C				180 °C			
SBS%		0	5	10	15	0	5	10	15	0	5	10	15	0	5	10	15
135 °C	0	-	S	S	S	S	S	S	S	S	N	S	S	S	S	S	S
	5		-	S	S	S	S	S	S	S	S	S	S	S	S	S	N
	10			-	S	S	S	S	S	S	S	S	S	S	S	S	S
	15				-	S	S	S	S	S	S	S	S	S	S	S	S
150 °C	0					-	S	S	S	S	S	S	S	S	S	S	S
	5						-	S	S	S	S	S	S	S	S	S	S
	10							-	S	S	S	S	S	S	S	S	S
	15								-	S	S	S	S	S	S	S	S
165 °C	0									-	S	S	S	N	S	S	S
	5										-	S	S	S	S	S	S
	10											-	S	S	S	S	S
	15												-	S	S	S	S
180 °C	0													-	S	S	S
	5														-	S	S
	10															-	S
	15																-

S: Significant. N: Non-significant.

**Table 7:** Statistical analysis results of viscosity for PG 64-22 with 12% processed oil as a function of SBS content (α = 0.05).

Viscosity		135 °C				150 °C				165 °C				180 °C			
SBS%		0	5	10	15	0	5	10	15	0	5	10	15	0	5	10	15
135 °C	0	-	S	S	S	S	S	S	S	S	N	S	S	S	N	S	S
	5		-	S	S	S	S	S	S	S	S	S	S	S	S	S	S
	10			-	S	S	S	S	S	S	S	S	S	S	S	S	S
	15				-	S	S	S	S	S	S	S	S	S	S	S	S
150 °C	0					-	S	S	S	N	S	S	S	N	N	S	S
	5						-	S	S	S	S	S	S	S	S	N	S
	10							-	S	S	S	S	S	S	S	S	N
	15								-	S	S	S	S	S	S	S	S
165 °C	0									-	S	S	S	N	S	S	S
	5										-	S	S	S	N	S	S
	10											-	S	S	S	S	S
	15												-	S	S	S	S
180 °C	0													-	S	S	S
	5														-	S	S
	10															-	S
	15																-

S: Significant. N: Non-significant.

**Table 8:** Statistical analysis results of G*/sin *δ* for PG 64-22 with processed oil as a function of SBS content (α = 0.05).

G*/Sin *δ*	SBS %	0	5	10	15
PG 64-22 + Processed Oil 6%	0	-	S	S	S
	5		-	S	S
	10			-	S
	15				-
PG 64-22 + Processed Oil 12%	0	-	N	S	S
	5		-	S	S
	10			-	S
	15				-

S: Significant. N: Non-significant.

**Table 9:** Statistical analysis results of G*/sin *δ* for RTFO aged PG 64-22 with processed oil as a function of SBS content (α = 0.05).

G*/Sin *δ*	SBS %	0	5	10	15
PG 64-22 + Processed Oil 6%	0	-	S	S	S
	5		-	S	S
	10			-	S
	15				-
PG 64-22 + Processed Oil 12%	0	-	N	S	S
	5		-	S	S
	10			-	S
	15				-

S: significant. N: Non-significant.

**Table 10:** Statistical analysis results of G*sin *δ* for RTFO + PAV aged PG 64-22 with processed oil as a function of SBS content (α = 0.05).

G*Sin *δ*	SBS %	0	5	10	15
PG 64-22 + Processed Oil 6%	0	-	S	S	S
	5		-	S	S
	10			-	S
	15				-
PG 64-22 + Processed Oil 12%	0	-	N	S	S
	5		-	S	S
	10			-	S
	15				-

S: Significant. N: Non-significant.

**Table 11:** Statistical analysis results of stiffness for RTFO + PAV aged PG 64-22 with processed oil as a function of SBS content at −12 °C (α = 0.05).

Stiffness	SBS %	0	5	10	15
PG 64-22 + Processed Oil 6%	0	-	N	S	S
	5		-	N	S
	10			-	S
	15				-
PG 64-22 + Processed Oil 12%	0	-	N	N	S
	5		-	N	S
	10			-	S
	15				-

S: Significant. N: Non-significant.

**Table 12:** Statistical analysis results of stiffness for RTFO + PAV aged PG 64-22 with processed oil as a function of SBS content at −24 °C (α = 0.05).

Stiffness	SBS %	0	5	10	15
PG 64-22 + Processed Oil 6%	0	-	S	S	S
	5		-	S	S
	10			-	S
	15				-
PG 64-22 + Processed Oil 12%	0	-	S	S	S
	5		-	S	S
	10			-	S
	15				-

S: Significant. N: Non-significant.

**Table 13:** Statistical analysis results of m-value for RTFO + PAV aged PG 64-22 with processed oil as a function of SBS content −12 °C (α = 0.05).

m-Value	SBS %	0	5	10	15
PG 64-22 + Processed Oil 6%	0	-	S	N	S
	5		-	S	S
	10			-	S
	15				-
PG 64-22 + Processed Oil 12%	0	-	S	N	N
	5		-	S	S
	10			-	N
	15				-

S: Significant. N: Non-significant.

**Table 14:** Statistical analysis results of m-value for RTFO + PAV aged PG 64-22 with processed oil as a function of SBS content −24 °C (α = 0.05).

m-Value	SBS %	0	5	10	15
PG 64-22 + Processed Oil 6%	0	-	S	S	S
	5		-	S	N
	10			-	S
	15				-
PG 64-22 + Processed Oil 12%	0	-	S	S	S
	5		-	N	S
	10			-	N
	15				-

S: Significant. N: Non-significant.

## Data Availability

The data used to support the findings of this study are included within the article.
